# Human iPSC-derived microglial cells protect neurons from neurodegeneration in long-term cultured adhesion brain organoids

**DOI:** 10.1038/s42003-024-07401-0

**Published:** 2025-01-09

**Authors:** Xianwei Chen, Guoqiang Sun, Lizhao Feng, E Tian, Yanhong Shi

**Affiliations:** 1 Department of Neurodegenerative Diseases, Beckman Research Institute of City of Hope, 1500 E. Duarte Rd, Duarte, CA 91010 USA; 2 State Key Laboratory of Cardiovascular Diseases and Medical Innovation Center, Shanghai East Hospital, School of Life Sciences and Technology, Frontier Science Center for Stem Cell Research, Tongji University, Shanghai, 200092 China

**Keywords:** Induced pluripotent stem cells, Stem-cell biotechnology

## Abstract

Brain organoid models have greatly facilitated our understanding of human brain development and disease. However, key brain cell types, such as microglia, are lacking in most brain organoid models. Because microglia have been shown to play important roles in brain development and pathologies, attempts have been made to add microglia to brain organoids through co-culture. However, only short-term microglia-organoid co-cultures can be established, and it remains challenging to have long-lasting survival of microglia in organoids to mimic long-term residency of microglia in the brain. In this study, we developed an adhesion brain organoid (ABO) platform that allows prolonged culture of brain organoids (greater than a year). Moreover, the long-term (LT)-ABO system contains abundant astrocytes and can support prolonged survival and ramification of microglia. Furthermore, we showed that microglia in the LT-ABO could protect neurons from neurodegeneration by increasing synaptic density and reducing p-Tau level and cell death in the LT-ABO. Therefore, the microglia-containing LT-ABO platform generated in this study provides a promising human cellular model for studying neuron-glia and glia-glia interactions in brain development and the pathogenesis of neurodegenerative diseases such as Alzheimer’s disease.

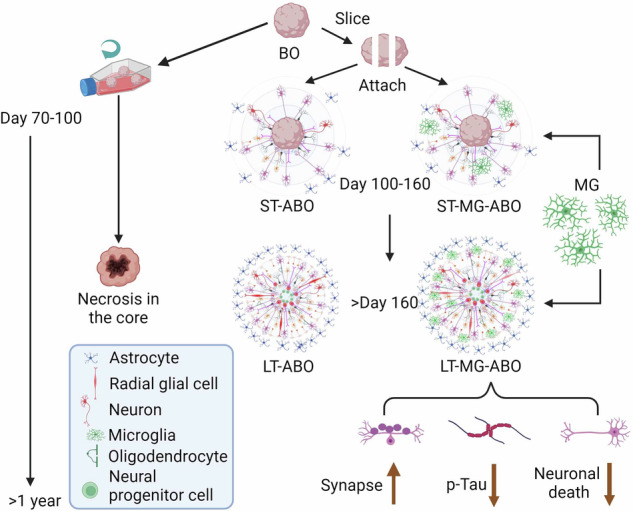

## Abstract (web summary)

Human iPSC-based modeling using microglia-containing long-term adhesion brain organoids reveals the role of microglia in supporting neuronal growth and function, as well as protecting neurons from neurodegeneration.

## Introduction

There is a high demand for the development of in vitro models of human brain development and diseases due to the inaccessibility of human brain tissues. The ability to differentiate human pluripotent stem cells (PSCs) into various neural lineage cells has allowed the establishment of self-organizing organoids that contain multiple brain cell types and can recapitulate various features of human brain development^1–3^. These organoid models can be maintained for months in culture to allow cellular and functional maturation^2,4^ and used to model human brain development in various ways^1,3,5–11^. However, because there is no vasculature and blood supply in current organoid system, prolonged maintenance of brain organoids in culture has been prohibited due to limited access to oxygen and nutrients at the core region of organoids. Accordingly, brain organoids within 5 months of differentiation have been used for most published studies^1,3,5–9^.

Strategies have been taken to enhance the survival of cells at the core and prolong the culture of organoids by increasing the accessibility of oxygen and nutrients, for example, by transplanting organoids into mouse brains to allow vascularization in vivo^12^. However, in vitro vascularization of brain organoids remains a challenge, thus alternative ways are needed to allow prolonged maintenance of brain organoids in vitro. Moreover, key brain cell types, including microglia, are lacking in most brain organoid models^1,3,5–9^, because microglia are mesoderm-derived, unlike other brain cell types, such as neurons and astrocytes that are ectoderm-derived.

Microglia are resident macrophages of the brain and have been shown to play important roles in brain development and disease^13^. Mutations in microglia-associated genes have been shown to be involved in the pathogenesis of various neurological disorders, including neurodegenerative diseases^14–16^. Moreover, single-cell-RNA-seq revealed that microglia can adopt disease-associated states^17–19^. Although increasing evidence has implicated microglia in both normal brain functions and the pathogenesis of neurological disorders, the precise roles of microglia in brain development and disease remains to be understood.

Elegant studies using animal models have helped us to gain much knowledge about microglia. However, recent studies have reported substantial species specificity of microglial transcriptome^20^. Due to species specificity, rodent microglia cannot recapitulate the properties of human microglia completely^21^. Therefore, it is important to study human brain development and disease using microglia of human origin. Increasing studies point to the importance of neuroimmune interactions in both normal brain functions and brain disorders. Therefore, human microglial models in the context of human neurons and astrocytes are needed to promote our understanding of brain development and pathologies related to brain-immune interactions.

In this study, we have developed an adhesion brain organoid (ABO) model that allows prolonged culture of human brain organoids (that can be maintained beyond a year). Moreover, the ABO system has allowed long-term survival of co-cultured microglia, mimicking the long-term residency of microglia in the brain. Using this microglia-containing ABO (MG-ABO) system, we showed that human iPSC-derived microglia were able to protect neurons from neurodegeneration in prolonged organoid cultures.

## Results

### Developing an adhesion brain organoid (ABO) system that can undergo prolonged culture

**Fig. 1:**
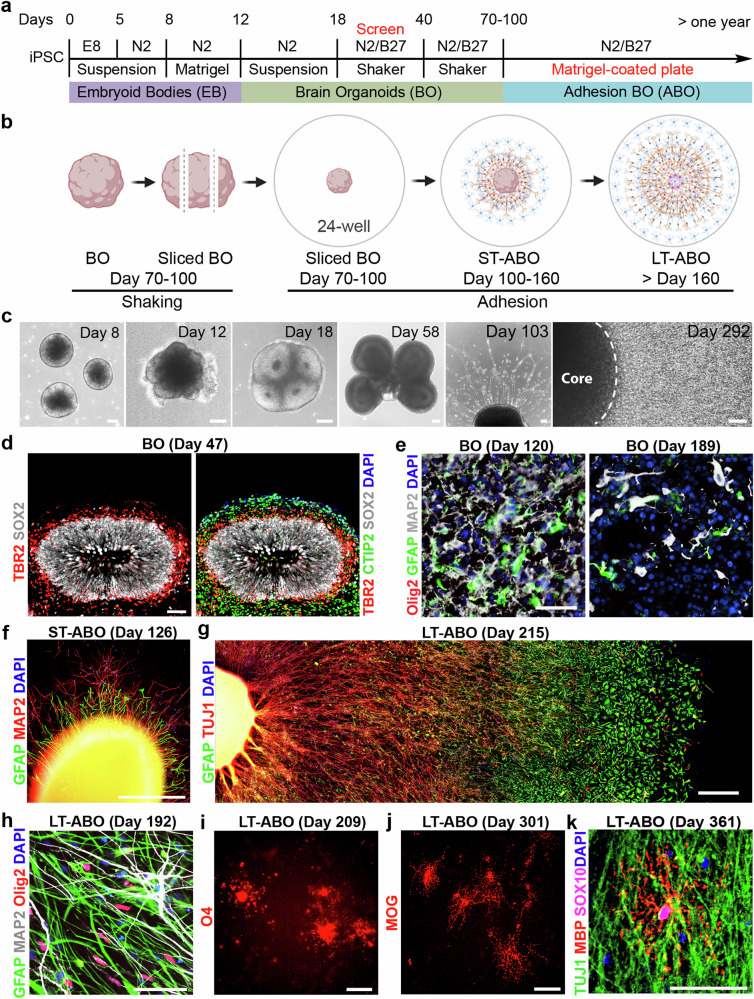
Development of the adhesion brain organoid (ABO) system. **a** Schematic illustration of brain organoid (BO) and ABO protocol. **b** Schematic illustration of the method for generating ABO. **c** Representative bright-field images of embryoid body (EB), BO, and ABO at different stages. ST-ABO: day 103; LT-ABO: day 292. The “core” area in the ABO represents the location of the originally seeded BO in a well of a 24-well plate. Scale bar, 100 µm. **d** Representative images of TBR2, SOX2 and CTIP2 staining in the BO at day 47. Scale bar, 50 µm. **e**, Representative images of Olig2, MAP2 and GFAP staining of BO at day 120 and 189. Scale bar, 50 µm. **f**, Representative images of GFAP and MAP2 staining of the ST-ABO at day 126. Scale bar, 500 µm. **g** Representative images of GFAP and TUJ1 staining in the LT-ABO at day 215. Scale bar, 500 µm. **h** Representative images of Olig2, MAP2 and GFAP staining of the LT-ABO at day 192. Scale bar, 50 µm. **i** Representative images of live-cell staining of O4 in the LT-ABO at day 209. Scale bar, 50 µm. **j** Representative images of live-cell staining of MOG in the LT-ABO at day 301. Scale bar, 50 µm. **k** Representative images of MBP, SOX10 and TUJ1 staining in the LT-ABO at day 361. Scale bar, 50 µm.

Human brain organoids have been increasingly used for modeling brain development and disorders. However, it is challenging to make prolonged culture of brain organoids due to limited access to oxygen and nutrients at the core region of organoids. To overcome this challenge, we developed an adhesion organoid protocol that allows long term culture of brain organoids in vitro built on recently published protocols for generating brain organoids with high consistency from studies of our own laboratory and others^3,5,8,22^. Specifically, we sliced brain organoids after day 70–100 when typical brain organoid layer structure was well-formed and cultured the organoid slices on Matrigel-coated plates to make adhesion organoid cultures (Fig. 1a–c).

The organoids at day 47 of differentiation exhibited characteristic layer structures, including the SOX2^+^ ventricular zone-like layer, the TBR2^+^ subventricular zone-like layer, and the CTIP2^+^ cortical layer (Fig. 1d). The organoids at day 70 to 100 of differentiation were sliced and then seeded onto Matrigel-coated 24-well plate for extended culture (Fig. 1a, b). The sliced organoids formed a core, with cells migrating out from the core and spreading to form the adhesion brain organoids (termed ABO in short) (Fig. 1b, day 103 and day 292).

The thickness of the ABOs decreases from the core to the outside region (Supplementary Fig. 1a). The approximate thickness of the core is around 300 µm (Supplementary Fig. 1b) and the outside region is composed of monolayer cells. Therefore, the ABOs have a 2.5D-like structure. The thickness of the core decreases slightly when more cells migrate out. The ABOs have some internal organization outside the core. For example, the astrocyte cell bodies stay together, and their processes show radial distribution. Moreover, cells migrate from the high-cell-density area to the low-cell-density area to form a density gradient.

The outward migration of neurons and astrocytes in the ABO was confirmed by staining for the neuronal marker MAP2 and the astrocyte marker GFAP (Fig. 1f). The distribution of astrocytes and neurons in the ABO at day 215 of differentiation was evaluated by staining for the neuronal marker βIII tubulin (TUJ1) and the astrocyte marker GFAP, which revealed neuronal distribution near the core followed by outward distribution of astrocytes (Fig. 1g). We could see a clear increase in the number of GFAP^+^ cells in the LT-ABOs compared to the ST-ABOs in the outer region (Fig. 1f, g, Supplementary Fig. 1c). We co-stained the GFAP^+^ cells in the ST-ABO and the LT-ABO with SOX2 and showed that the majority of the GFAP^+^ cells are SOX2^-^GFAP^+^ astrocytes and a small population of the GFAP^+^ cells are SOX2^+^GFAP^+^ (Supplementary Fig. 1d), suggesting that a small portion of the GFAP^+^ cells are radial glial cells. We detected increased percentage of SOX2^-^GFAP^+^ astrocytes in GFAP^+^ cells in the LT-ABO compared to the ST-ABO, but decreased percentage of SOX2^+^GFAP^+^ cells in the LT-ABO, although the changes did not reach statistical significance (Supplementary Fig. 1d).

We cultured brain organoids in the classic suspension culture or the adhesion culture we developed in parallel for side-by-side comparison. We noticed that the MAP2^+^ neurons and the GFAP^+^ astrocytes were reduced in the suspension cultured organoids at day 189, compared to that in the suspension cultured organoids at day 120 (Fig. 1e), indicating that the suspension cultured organoids were less healthy when cultured for a long time. In contrast, the adhesion cultured organoids remained healthy even after prolonged culture (Fig. 1c, g–k). These results indicate that the adhesion organoid platform allows us to generate healthy organoids for prolonged culture.

We defined the ABO at day 70–100 to day 160 of differentiation as short-term ABO (ST-ABO) and the ABO beyond day 160 of differentiation as long-term ABO (LT-ABO). The ST-ABO usually covers <50% of the surface area of one well in a 24-well plate with limited astrocytes migration (Fig. 1b, c, f). In contrast, the LT-ABO usually covers >50% of the surface area of one well in a 24-well plate with a large number of astrocytes migrating out (Fig. 1b, c, g). The LT-ABO could be cultured without the need of a shaker for more than a year (Fig. 1a, b). These results together indicate that the LT-ABO is an organoid platform that is easy to handle and allows prolonged culture.

### The ABO organoids can support natural oligodendroglial differentiation

Unlike neurons and astrocytes, oligodendroglial lineage cells, including oligodendrocyte progenitor cells (OPC) and oligodendrocytes (OL), were not observed in the classic suspension cultured brain organoids at day 120 and day 189 of differentiation (Fig. 1e). Surprisingly, the oligodendrocyte transcription factor 2 (Olig2)-positive OPCs could be detected in the ST-ABO from day 141 after differentiation (Fig. S2). Increasing number of OLIG2^+^ OPCs were observed at the outer region of the LT-ABO at day 192 (Fig. 1h). The O4^+^ OPCs were detected in the LT-ABO by live cell staining at day 209 (Fig. 1i). In addition, the myelin oligodendrocyte glycoprotein (MOG)-positive and myelin basic protein (MBP)-positive OLs were observed in the LT-ABO after >300 days of differentiation (Fig. 1j, k). Although we did not quantify the percentage of OLIG2^+^ cells in the ABO because we did not collect a sufficient number of ABO at each stage, our detection of OLIG2^+^ or O4^+^ OPCs and MOG^+^ or MBP^+^ oligodendrocytes in the ABO indicates that adhesion cultured organoids can better support oligodendroglial differentiation than suspension cultured organoids. It is worth noting that the differentiation time line of Olig2^+^ OPCs, O4^+^ pre-OLs, and MBP^+^ OLs in ABO is consistent with the developmental time line of OPC/OL in human brains, in the order of expansion peaks of OPCs (15–20 gestational weeks) and pre-OLs (30–40 gestational weeks) followed by the emergence of the MBP^+^ OLs (~1 postnatal month)^23^. These results indicate that the ABO platform established in this study could recapitulate the natural oligodendroglial differentiation process occurred during human brain development.

### The LT-ABO can support long-term microglial co-culture

Because microglia are mesoderm-derived, whereas brain cells including neurons and astrocytes are ectoderm-derived, microglia were not intrinsically developed in most brain organoid models^1,3,5–9^ with one exception in which the organoids were generated using a protocol that deviated from the standard way to allow mesodermal development^24^. To facilitate our understanding of brain-immune interactions, we attempted to generate brain organoids with microglia by co-culturing human iPSC-derived microglia with brain organoids of ectoderm origin as described^6,7^.

**Fig. 2:**
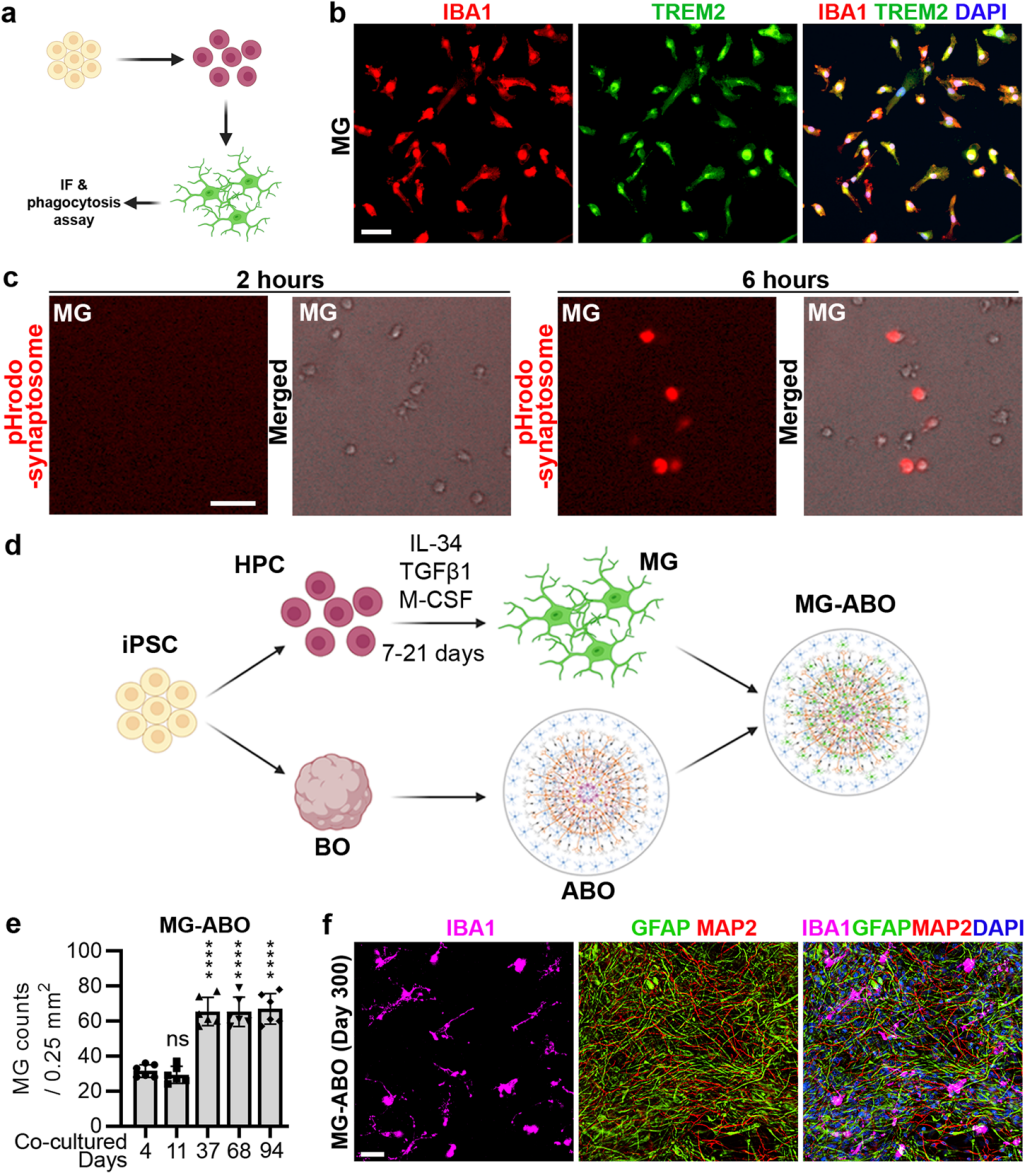
Generation of microglia (MG)-containing ABO (MG-ABO) by co-culture with MG. **a** Schematic illustration of differentiation of human iPSCs into MG. **b** Representative images of IBA1 and TREM2 staining in human iPSC-derived MG at day 30 of differentiation. Scale bar, 50 µm. **c** Human iPSC-derived MG (at day 29 of differentiation) engulfed pHrodo-labeled synaptosomes within 6 h. Scale bar, 50 µm. **d** Schematic illustration of the method for generating MG-ABO. **e** Quantification of the GFP-MG density in MG-ABO at regions around but outside of the “core”. *n* = 6 images for each time point. Error bars are SD of the mean. All comparisons were performed between MG density at 4 day co-culture and that of any other co-culture time. ns: not statistically significant. ****p < 0.0001 by unpaired *t*-test. **f** Representative images of IBA1, MAP2 and GFAP in MG-ABO at day 300 of organoid differentiation (with MG co-culture for 82 days). Scale bar, 50 µm.

Human iPSCs were differentiated into microglia as described^25^. The iPSC-derived microglia expressed the microglial markers IBA1 and TREM2 (Fig. 2b). Moreover, these microglia were functional. They were able to phagocytose pHrodo-labeled synaptosomes effectively after 6-hour (hr) incubation with the pHrodo-synaptosomes (Fig. 2c). We then co-cultured the human iPSC-derived microglia with suspension cultured organoids. However, similar to the published studies^6,7^, the co-cultured microglia were only able to be maintained for a short period of time in suspension cultured organoids (Supplementary Fig. 3).

To allow prolonged co-culture of microglia with brain organoids to better mimic long-term residency of microglia in the human brain, we took advantage of our ABO system that has a unique advantage for co-culturing with microglia. The ABO has an open interface that allows easy seeding and integration of microglia into the ABO. To facilitate microglial tracking, we knocked in a GFP reporter into the AAVS1 safe harbor site in human iPSCs using TALEN-based gene editing^26^ and differentiated the GFP-labeled iPSCs into microglia. We then seeded GFP-labeled human iPSC-derived microglia (GFP-MG) onto the ABO uniformly and monitored microglial survival in the co-culture with the ABO using the GFP fluorescence. The density of GFP-MG at the area surrounding the core region was monitored for up to 94 days. We found that the GFP-MG density was first increased and then maintained for the time when we monitored (Fig. 2e), indicating that microglia can survive well for a long time (>3 months) in the ABO. The microglia-containing ABO was termed MG-ABO. Immunostaining analysis revealed that microglia were well incorporated into the ABO. The MG-ABO contained the IBA1^+^ microglia together with the MAP2^+^ neurons and GFAP^+^ astrocytes (Fig. 2f). Taken together, these results indicate that the ABO platform can support long-term microglial survival.

**Fig. 3:**
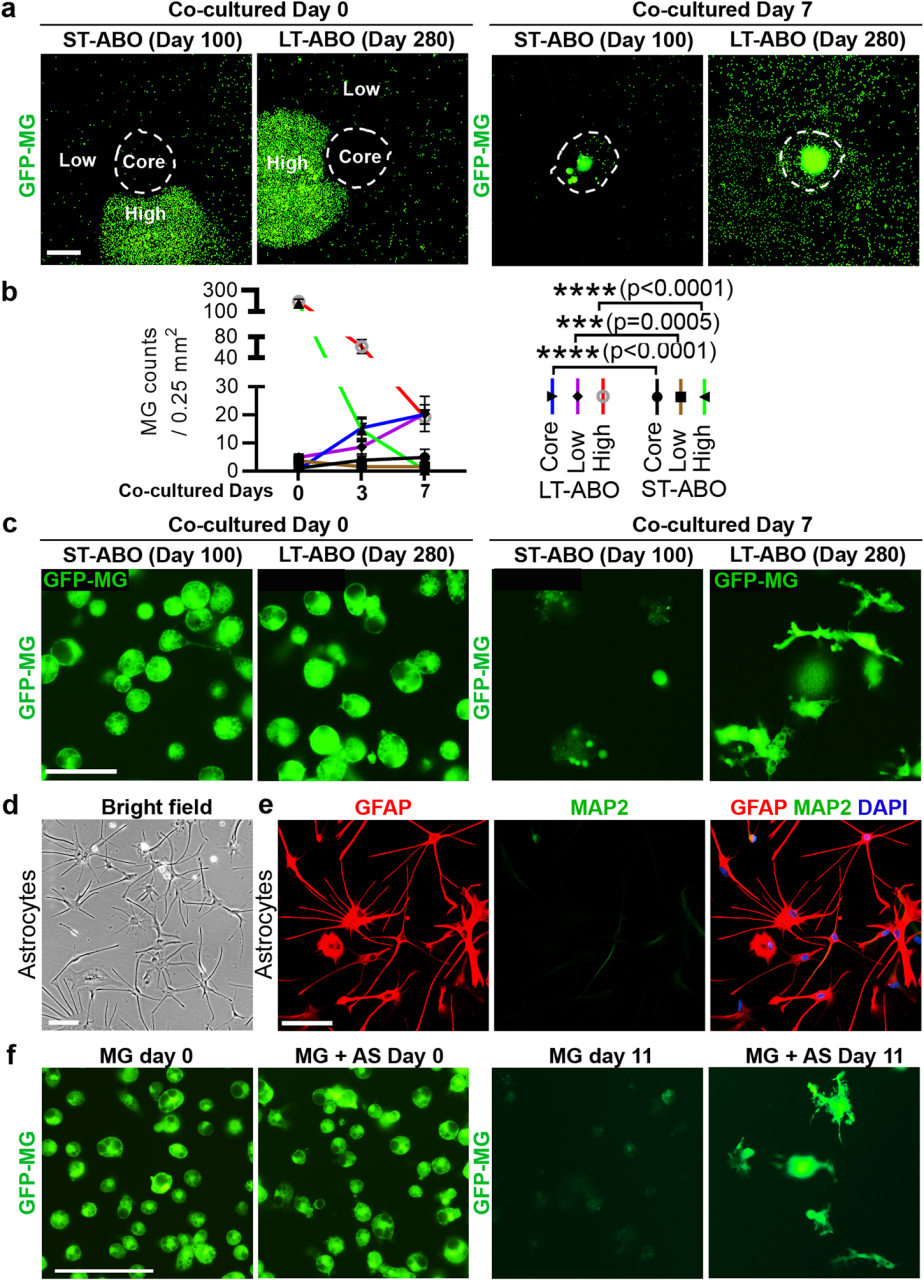
Astrocytes (AS) support microglia survival and ramification in the LT-ABO. **a** Co-culture of GFP-MG with the ST-ABO or the LT-ABO. GFP-MG were seeded at a defined area outside but around the core region, which is labeled as the “High” area, representing an area with high GFP-MG density. The remaining outer area is labeled as the “Low” area, representing an area with low GFP-MG density. Scale bar, 500 µm. **b** Quantification of GFP-MG density in co-cultures with the ST-ABO or the LT-ABO at the “Core”, the “High”, or the “Low” regions. *n* = 6 images for each time point. Error bars are SD of the mean; ***p < 0.001 and ****p < 0.0001 by two-way ANOVA test. **c** Representative images of GFP-MG in co-cultures with the ST-ABO or the LT-ABO. Scale bar, 50 µm. **d** A representative bright field image of astrocytes isolated from the LT-ABO. Scale bar, 100 µm. **e** Representative images of GFAP and MAP2 staining of astrocytes isolated from the LT-ABO. Scale bar, 100 µm. **f** Representative images of GFP-MG cultured alone or co-cultured with astrocytes for 11 days. Scale bar, 50 µm.

To determine if the short-term (ST) ABO and the long-term (LT) ABO could both support microglial survival, GFP-MG were seeded onto the ST-ABO or the LT-ABO at a defined region around the “core” area (Fig. 3a, b). The majority of GFP-MG was located in a small area adjacent to the core region of the ABO. This small area was defined as the “high” area because of the high density of GFP-MG. The remaining area outside of the core has very low GFP-MG density, thus was defined as the “low” area. The core of the ABO also exhibited low GFP-MG density and was labeled as the “core” area (Fig. 3a, b).

The microglia-containing ST-ABO and LT-ABO were termed ST-MG-ABO and LT-MG-ABO, respectively. After 7 days of co-culture in the organoid medium, the number of GFP-MG in the ST-MG-ABO was reduced dramatically at both the “high” and the “low” areas and was slightly increased at the “core” area. On the other hand, the overall GFP-MG density at all three defined areas in the LT-MG-ABO was significantly higher than that in the ST-MG-ABO after 7 days of co-culture (Fig. 3a, b). The GFP-MG density at the “high” and the “low” regions exhibited opposite change after 7 days of co-culture in the LT-MG-ABO, indicating that GFP-MG could migrate from the high-density area to the low-density area (Fig. 3a, b). In addition, more GFP-MG migrated to the core of the LT-MG-ABO than that to the core of the ST-MG-ABO (Fig. 3a, b). After 7-day co-culture, the number of GFP-MG in the ST-MG-ABO was reduced substantially (Fig. 3a–c), suggesting cell death of GFP-MG in these organoids. Conversely, the GFP-MG in the LT-MG-ABO not only maintained their abundance but also developed processes (Fig. 3a–c), indicating that the LT-ABO culture could not only support microglial survival but also enhance microglia ramification. The density of the microglia in the LT-MG-ABO is around 13%, which is close to the microglial density in the brain (about 10%)^27^ (Supplementary Fig. 4a, b). A small portion of the microglia in the LT-ABOs are proliferative as revealed by Ki67 staining (Supplementary Fig. 4a, c). These results together indicate that the LT-ABO can support microglia sustainability better than the ST-ABO.

**Fig. 4:**
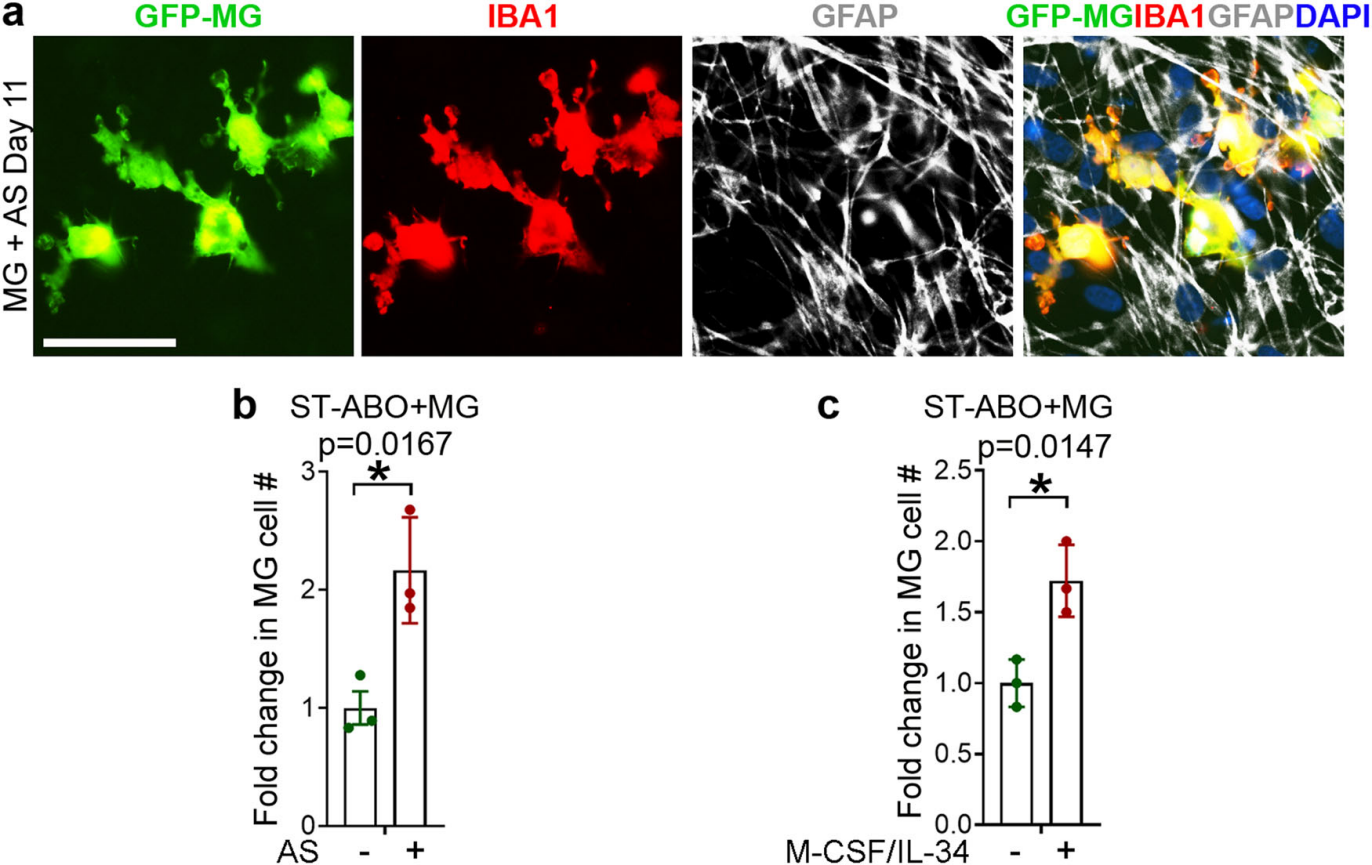
Co-culture of microglia with astrocytes or ST-ABO plus astrocytes. **a** Representative images of GFP-MG and IBA1/GFAP staining of microglia co-cultured with astrocytes isolated from the LT-ABO for 11 days. Scale bar, 50 µm. **b** The fold change of microglial cell number (#) in co-culture with the ST-ABO with or without astrocytes. GFP-MG cells were co-cultured for 20 days with the ST-ABO alone (- AS) or the ST-ABO plus astrocytes (+ AS) isolated from the LT-ABO. The fold change in the MG cell number was calculated relative to the MG cell number in co-culture with the ST-ABO alone (- AS). **c** The fold change of microglial cell number (#) in co-culture with the ST-ABO treated with or without microglia-supporting factors including M-CSF and IL-34. GFP-MG cells were co-cultured with the ST-ABO treated with or without microglia-supporting factors including M-CSF and IL-34. The fold change in the MG cell number was calculated relative to the MG cell number in co-culture with the ST-ABO without treatment (- M-CSF/IL-34). *n* = 3 organoids for each condition. Error bars are SD of the mean; *p < 0.05 by unpaired *t*-test for panels (**b**, **c**).

Comparing the cellular composition of the ST-ABO and the LT-ABO revealed that the LT-ABO contained much more abundant GFAP+ astrocytes (Fig. 1f, g, and Supplementary Fig. 1c), consistent with previous studies showing that astrocytes are required for microglial survival and ramification^28,29^. To test the idea that astrocytes in the LT-ABO can promote microglial survival and ramification, we isolated astrocytes from the LT-ABO. The astrocytes isolated from the LT-ABO showed typical star-shape morphology with multiple processes and were GFAP-positive and MAP2-negative (Fig. 3d, e). These astrocytes were used to co-culture with GFP-MG. Culture of microglia alone was included as a negative control. Co-culture with astrocytes isolated from the LT-ABO supported microglial survival and ramification in a manner similar to the co-culture with the LT-ABO, whereas the microglia only culture failed to survive well in the same medium (Fig. 3f). The presence of both astrocytes and microglia in the co-culture was confirmed by co-staining of the co-culture for the astrocyte marker GFAP and the microglial marker IBA1 as well as the GFP-MG fluorescence (Fig. 4a). Next, we applied microglia to the ST-ABO alone or the ST-ABO co-cultured with astrocytes isolated from the LT-ABO. Co-culture of the ST-ABO with astrocytes isolated from the LT-ABO led to increased number of microglial cells in the ST-ABO, compared to that in the ST-ABO without astrocyte co-culture (Fig. 4b), further supporting the idea that astrocytes from the LT-ABO can support microglial survival. Adding astrocyte-derived factors that have been shown to promote microglial survival including M-CSF and IL-34^28^ to the ST-ABO also promoted microglial survival (Fig. 4c). These results together indicate that the LT-ABO can support microglial survival and ramification, presumably because of the presence of abundant astrocytes in the LT-ABO that can secret microglia-supporting factors.

### Microglia in the LT-ABO can protect neurons from neurodegeneration

**Fig. 5:**
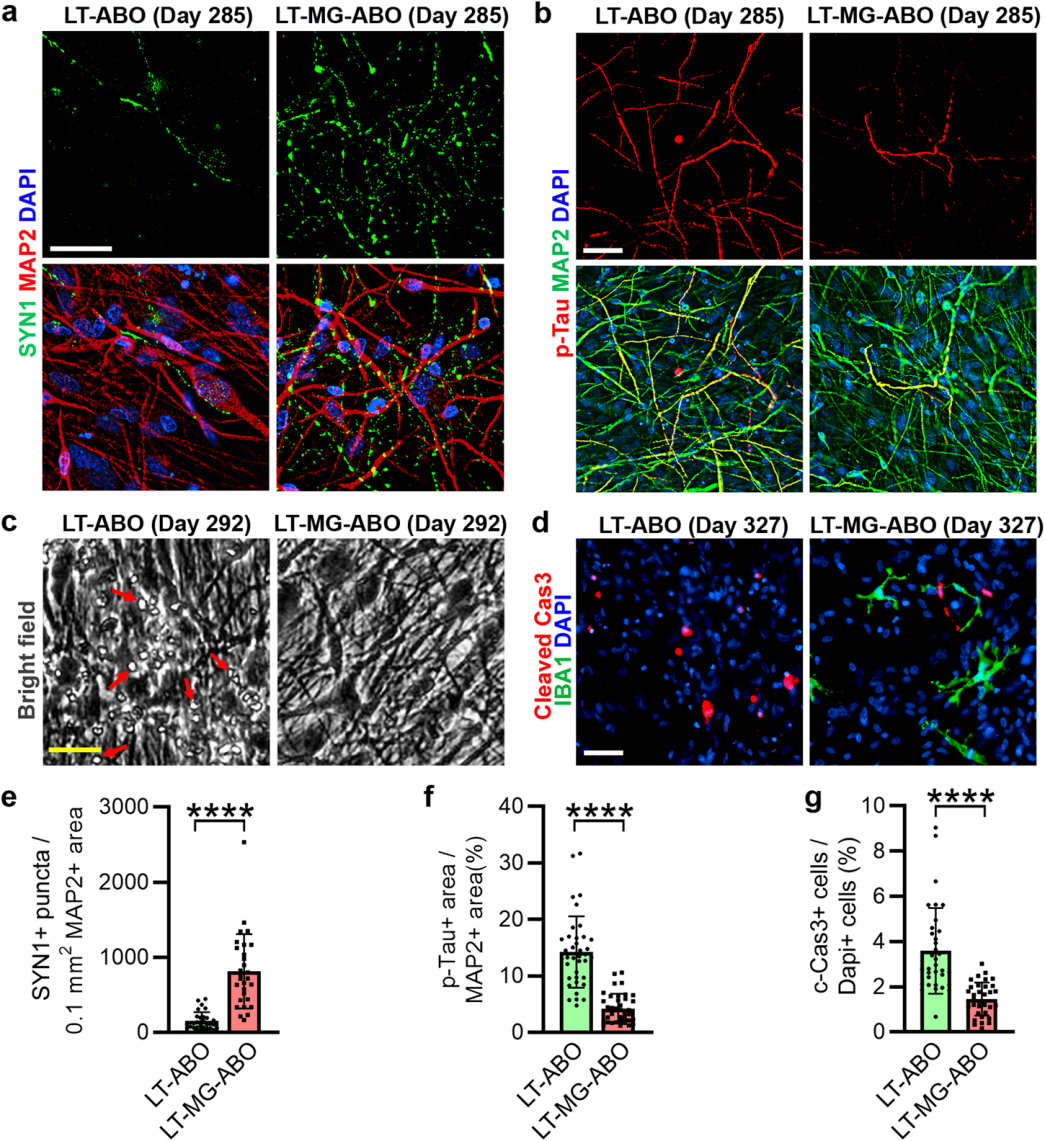
Microglia (MG) protect neurons from degeneration in LT-ABO. **a** Representative images of SYN1 and MAP2 staining in the LT-ABO and the LT-MG-ABO at day 285 of organoid differentiation (with MG co-culture for 125 days). Scale bar, 50 µm. **b** Representative images of p-Tau and MAP2 staining in the LT-ABO and the LT-MG-ABO at day 285 (with MG co-culture for 125 days). Scale bar, 50 µm. **c** Representative bright-field images of the LT-ABO and the LT-MG-ABO. Arrows point to cell debris. Scale bar, 50 µm. **d** Representative images of cleaved caspase 3 (c-Cas3) and GFAP staining in the LT-ABO and the LT-MG-ABO at day 327 (with MG co-culture for 83 days). Scale bar, 50 µm. **e**–**g** Quantification of SYN1+ synaptic puncta, p-Tau and c-Cas3 levels in the LT-ABO and the LT-MG-ABO. *n* = 29 images (SYN1), 38 images (p-Tau) and 34 images (c-Cas3) from 3 individual organoids per group with 6–15 images for each organoid for each condition. Error bars are SD of the mean; ****p < 0.0001 by unpaired *t*-test.

**Fig. 6:**
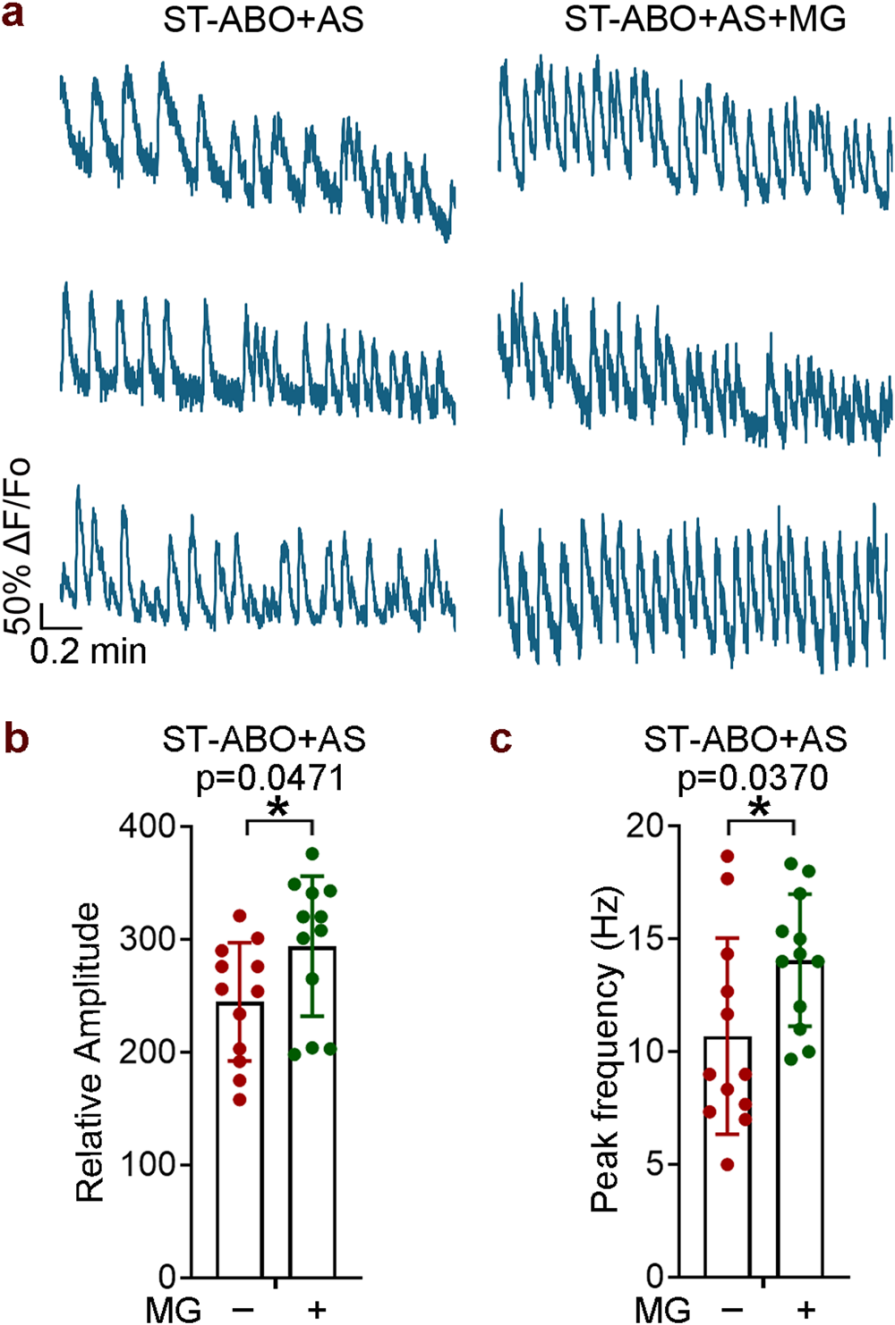
Calcium signaling of the ST-ABO co-cultured with astrocytes alone or astrocytes and microglia. **a** Calcium imaging of the ST-ABO co-cultured with astrocytes isolated from the LT-ABO alone (ST-ABO + AS) or astrocytes and microglia (ST-ABO + AS + MG). **b**, **c** The amplitude and frequency of calcium oscillation in the ST-ABO co-cultured with astrocytes alone (ST-ABO + AS) or astrocytes and microglia (ST-ABO + AS + MG). *n* = 4 organoids for each condition with 3 regions of interests selected for each organoid. Error bars are SD of the mean; *p < 0.05 by unpaired *t*-test for **b**, **c**.

It has been shown that microglia play important roles in brain development and disease^13^. However, the precise role of microglia in the brain through brain-immune interactions remains to be understood. Taking advantage of the LT-MG-ABO model that we established in this study, we determined the effects of microglia on the LT-ABO after 83 days to 125 days of co-culture. Studies have revealed bidirectional effects of microglia on synapses by promoting synapse formation^30–32^ and facilitating synapse elimination^33^. We noticed that the number of synapsin (SYN1)^+^ synaptic puncta was relatively low in the LT-ABO without microglial co-culture, but increased significantly in the LT-MG-ABO that contained microglia (Fig. 5a, e). This result suggests that microglia can promote synapse formation or prevent synapse deterioration in the LT-ABO. Moreover, we co-cultured the ST-ABO with astrocytes isolated from the LT-ABO to mimic the LT-ABO. We detected enhanced calcium signaling, including increased amplitude and oscillation frequency in the ST-ABO co-cultured with astrocytes and microglia, compared to that in the ST-ABO co-cultured with astrocytes alone (Fig. 6), indicating that microglia can promote neuronal activity.

It has been shown that elevated level of hyperphosphorylated Tau (p-Tau) is a pathological feature of neurodegenerative diseases, including Alzheimer’s disease^34–36^. Of interest to us, we found that the level of p-Tau was reduced substantially in the LT-MG-ABO that contains long-term co-cultured microglia, compared to that in LT-ABO without microglia (Fig. 5b, f), indicating that microglia can protect neurons from the accumulation of p-Tau. Furthermore, we found debris with a size smaller than that of a cell in the LT-ABO without microglia. The debris was reduced dramatically in the LT-MG-ABO (Fig. 5c), presumably due to the clearance by microglia. Accordingly, the cleaved caspase 3 (c-Cas3)-positive cells were reduced substantially in the LT-MG-ABO, compared to that in the LT-ABO (Fig. 5d, g), indicating reduced cell death in the LT-MG-ABO that contained microglia. These results together indicate that microglia can have robust neuroprotective effects on LT-ABO by increasing neuronal synaptic density and reducing p-Tau level and cell death in the co-cultured organoids.

## Discussion

Human brain organoids are self-organizing tissues mimicking the structure and cellular composition of the human brain^37^. The combination of the human iPSC technology^38,39^ with the brain organoid platform has allowed modeling of both human brain development and brain disorders^1,3,5–11,40,41^. However, current brain organoid models lack the vasculature system and the blood, leading to reduced viability of cells at the core of large organoids due to the limited supply of oxygen and nutrients^3,42–44^. Brain organoids cultured in suspension can grow to a size of 3 to 4 mm in diameter. However, cells in the core region of suspension organoids have limited access to oxygen and nutrients, thus start to die after prolonged culture. Creative ways have been developed to address this challenge by using the air-liquid interface culture^45^ or slicing organoid technique^22^ to expose the core region of the organoids to oxygen and culture media, which has allowed improved cell survival and more sustained organoid culture. In this study, we combined the advantage of the air-liquid interface and the slicing protocols and simplified the procedure, which has allowed us to develop an adhesion organoid system that is very easy to handle. The ABO system established in this study allows prolonged culture of brain organoids for over a year without the need for repeated slicing and shaking and the adhesion organoids are healthier than the suspension culture at similar time points after differentiation. The ABO platform is reproducible and can generate organoids in a consistent manner with minimal organoid-to-organoid variations. This strategy may also be used to make long term organoids of other organs beyond the brain.

Because microglia are mesoderm-derived, microglia are not differentiated naturally from iPSCs in standard brain organoid culture conditions that favor ectoderm differentiation, therefore do not emerge in brain organoids spontaneously like neurons and astrocytes that are ectoderm-derived. Consequently, microglia are lacking in most brain organoid models^1,3,5–9^. Because microglia play important roles in both normal brain functions and the pathogenesis of neurological disorders, it is critical to integrate microglia to the brain organoid system to better study brain development and disease. One way to solve this problem is to transplant microglia into brain organoids. Indeed, Popova et al. have added microglia into brain organoids and shown that microglia promoted neural network maturation in the organoids^6^. However, microglia could not last long in this model. The number of microglia reduced dramatically within 5 weeks of co-culture^6^. Similarly, we found that microglia could not survive for a long time when co-cultured with suspension brain organoids or ST-ABO. However, our LT-ABO platform could support long time survival of microglia beyond 120 days of co-culture. Moreover, microglia exhibited increased branches at the organoid-covered area but not at the non-organoid-covered area when co-cultured with the LT-ABO, supporting the idea that the LT-ABO can not only support microglial survival but also promote microglia ramification.

One limitation of the study is that we were not able to quantify the percentage of neurons, astrocytes, and oligodendroglial lineage cells in the ABO precisely because the core of the ABO is dense with multiple layers of cells, which makes it difficult to accurately determine the total number of cells in the core, thus the total number of cells in the ABO. Because the cell density in the outside region is lower, we were able to quantify the percentage of cells in the outside region of the ABO.

One major difference between the ST-ABO and the LT-ABO is the dramatically increased astrocyte population in the LT-ABO. Astrocytes are the main supporting cells within the central nervous system^46^. It has been shown that astrocytes promote microglial survival and ramification through astrocyte-derived factors^28,29^. Indeed, we found that astrocytes isolated from the LT-ABO could support microglial survival and ramification in a manner similar to the LT-ABO, confirming that astrocytes can support microglial growth and ramification. The high abundance of astrocytes in the LT-ABO provides a plausible explanation as to why microglia can last for long term in the LT-ABO but not in the ST-ABO or suspension organoids that do not contain a large number of astrocytes. Thus, our study provides a possible solution to improve microglial survival in organoids by enhancing astrogenesis in organoids.

Astrocytes and microglia are key glial cell types in the brain that maintain the homeostasis and support the function of the brain^47^. The absence of either astrocytes or microglia can impair neuronal functions in the brain severely^15,29,48^. In this study, we developed an ABO platform with a large population of astrocytes and long-lasting microglia, thus providing an ideal platform for evaluating long-term effects of microglia on brain development and disease. Indeed, we found that microglia could robustly protect neurons from neurodegeneration in the LT-MG-ABO. Besides the crosstalk between astrocytes and neurons or microglia and neurons, the crosstalk between microglia and astrocytes also play critical roles during brain development and pathological progression^49^. Therefore, the LT-MG-ABO platform generated in this study provides a promising human cellular model for studying the crosstalk among astrocytes, microglia and neurons and the pathological impact of dysregulated neuron-glia and glia-glia interactions on neurodegenerative diseases, including Alzheimer’s disease.

## Methods

### Human iPSC derivation

The iPSCs was reprogrammed from human fibroblasts, which were purchased from Coriell (AG14048 and AG10884) and reprogrammed as described previously through episomal reprogramming using episomal plasmids expressing OCT4, SOX2, L-MYC, KLF4, shp53, and EBNA1 (Addgene plasmids pCXLE-hSK, pCXLE-hUL, pCXLE-hOCT3/4-shp53-F, and pCXWB-EBNA1)^5,50^. Specifically, human fibroblast cells were electroporated with the reprogramming factors using 4D Nucleofector (Lonza) and seeded into 6-well plates coated with 1:100 diluted Matrigel (Corning) and maintained in E8 medium (Invitrogen). The iPSCs were confirmed to be karyotypically normal and pluripotent via teratoma formation or in vitro trilineage differentiation. In addition, all lines are routinely tested to confirm no mycoplasma contamination. The iPSCs were maintained at 37 °C in Matrigel-coated 6-well plates with daily medium change and passaged every 3–4 days using 0.5 mM EDTA treatment and manual dissociation.

### Generation of GFP-labeled iPSCs using TALEN editing

The GFP-labeled iPSCs were generated by TALEN-mediated gene editing. The hAAVS1 TALEN Right (Addgene#52342), hAAVS1 TALEN Left (Addgene#52341) and AAVS1-CAG-hrGFP (Addgene#52344) donor vectors were delivered via nucleofection into iPSCs^51^. The transfected iPSCs were seeded as single cells. The single cell-derived clones with GFP were picked and expanded for microglia differentiation. The hAAVS1 TALEN Right, hAAVS1 TALEN Left and AAVS1-CAG-hrGFP vectors were gifts from Dr. Su-Chun Zhang.

### Generation of BO from human iPSCs

BO was generated from AG14048 iPSCs using our published protocol^5,8^ with some modifications, as shown in Fig. 1a. Briefly, on day 0, human iPSCs were dissociated with EDTA, and the iPSC colonies were seeded into a 6-well suspension plate to form embryoid bodies (EBs) in E8 medium with 5 μM ROCK inhibitor Y-27632. On day 1, EBs were lifted manually by blowing gently using a 1 mL pipettor and cultured in fresh E8 medium without Y-27632. From day 1 to day 5, EBs were cultured in E8 medium with half medium change daily. On day 5, E8 medium was replaced by neural induction medium (NIM) containing DMEM/F12, N2, minimum essential medium NEAA (MEM-NEAA), and 2 μg/ml Heparin. From day 5 to day 8, EBs were cultured in NIM with half medium change daily. On day 8, EBs were embedded in Matrigel in NIM in a 12-well suspension plate and incubated at 37°C for 4 h, followed by adding 2 mL NIM. From day 8 to day 12, the BOs were cultured in NIM medium with half medium change daily. On day 12, BOs were lifted and transferred to a new suspension 6-well plate with NIM. From day 12 to day 20, the BOs were cultured in NIM with half medium change daily. On day 18–20, the BOs were transferred to a T25 suspension culture flask in the BO differentiation medium containing DMEM/F12, 2.5 μg/ml Insulin, Glutamax, MEM-NEAA, 3.5 μl/L (V/V) 2-mercaptoethanol, N2, and B27 on an Orbi-Shaker (Benchmark Scientific). Medium was changed every 3 days. To generate BOs with good reproducibility and homogeneity, a screening was performed from day 20 to day 40 and only the BO pass the criteria^5^ were kept for further culture. Form day 50 to day 70, the BOs with a diameter >1.5–2.0 mm could be cut into two halves to reduce necrosis at the core.

### Generation of ABO

To generate ABO, each BO at day 70–100 of differentiation was sliced into three sections as shown in Fig. 1c. Then, the sliced BOs were seeded into a 24-well plate with one section per well (coated with Matrigel one day before). The ABOs were cultured in BO differentiation medium with half medium change each 2-3 days.

### Isolating astrocytes from ABO

Astrocytes were isolated from ABO after Accutase digestion of ABO for about 30–60 min at 37°C, followed by centrifugation at 200 g for 2 min. The isolated astrocytes were seeded into Matrigel-coated plates and cultured in the total BO medium. Four to eight hours after plating, cells were gently washed using a pipette to remove dead cells and loose attached cells. The astrocytes were split once every month.

### Differentiation of iPSCs to hematopoietic progenitor cells (HPCs)

AG14048 and AG10884-GFP iPSCs were differentiated to HPCs using STEMdiff™ Hematopoietic Kit (Cat. # 05310) following the manufacturer’s guidance.

### Differentiation of HPCs to microglia

AG14048 and AG10884-GFP HPCs were differentiated to microglia following the published protocol^25^ with some modifications. The HPCs were seeded onto Matrigel-coated 12-well plate with 0.1 to 0.2 million cells per well in microglia differentiation medium containing DMEM/F12, 2X insulin-transferrin-selenite, 2X B27, 0.5X N2, 1X glutamax, 1X MEM non-essential amino acids, 400 μM monothioglycerol, supplemented with 5 μg/mL insulin, 100 ng/mL IL-34, 50 ng/mL TGFβ1, and 25 ng/mL M-CSF before use. After 7 to 21 days of differentiation, the microglia were used for co-culture experiments.

### Co-culturing microglia with BO or astrocytes

iPSC-derived microglia were used for co-culturing with conventional BO or ABO. For co-culturing microglia with ABO, two ways were used: (1) To seed microglia to a whole ABO, 0.02 million microglia were resuspended in brain organoid medium and then gently added to the ABO in a 24-well plate with one ABO per well. (2) To seed microglia in a defined region of ABO, 0.02 million microglia were resuspended in brain organoid medium and seeded very gently at a defined region of ABO in a 24-well plate under a dissection microscope. The plate was transferred back to a tissue culture incubator very gently after the microglia settled down to the bottom of the plate.

Microglial cells were added to the LT-ABOs around 3 months and to the ST-ABOs around 1 month after cutting and adhering to the plates. After adhering to the plates, organoids kept growing and differentiating. The section wound from the splitting should have been gone already when microglial cells were added. For co-culturing microglia with conventional BO, 4 × 10^5^ microglia were added to one conventional BO around day 50 of BO differentiation in a U-bottom 96-well plate. BO were co-cultured with microglia for overnight with one BO per well in the 96-well plate. The next day, organoids were transferred into 25 ml flasks using a pipette. These organoids were maintained on an orbital shaker in brain organoid medium until analysis. For co-culturing with astrocytes, 0.02 million microglia were co-cultured with or without 0.3 million astrocytes in one well of a Matrigel-coated 24-well plate in the total BO medium.

### Immunostaining of suspension cultured BO

The suspension cultured BOs were washed with phosphate-buffered saline (PBS) and fixed with 4% paraformaldehyde (PFA) at 4 °C for overnight, followed by incubation in 30% sucrose at 4 °C for overnight. Then the BOs were embedded in OCT and sectioned at a thickness of 16–20 µm using the Leica CM3050S cryostat. Cryosections were washed in PBS to remove OCT and incubated with 4% PFA for 10 min at room temperature (RT). Next, the sections were blocked in PBS with 3% donkey serum and 0.1% Triton X-100 at RT for 1 h. The sections were then incubated at 4 °C for overnight with primary antibodies diluted in PBS with 3% donkey serum and 0.01% Triton X-100. After washing with PBS with 0.01% Triton X-100, the sections were incubated at RT for 1 h with secondary antibodies diluted in PBS with 3% donkey serum and 0.01% Triton X-100. After washing with PBS with 0.01% Triton X-100, the sections were stained with DAPI before mounting. Images were obtained using a Carl Zeiss LSM700 confocal microscope or Nikon Eclipse Ti2 fluorescence microscope imaging system. The detailed antibody information is listed in Supplementary Table 1.

### Immunostaining of ABO

The adhesion cultured ABO washed with PBS and then fixed with 4% PFA for 1–2 h at 37°C. The fixed ABO were blocked in PBS with 3% donkey serum and 0.1% Triton X-100 for 1 h at 37°C, and then incubated at 37 °C for 1 h with primary antibodies diluted in PBS with 3% donkey serum and 0.01% Triton X-100. After washing with PBS with 0.01% Triton X-100, the ABOs were incubated at 37 °C for 1 h with secondary antibodies diluted in PBS with 3% donkey serum and 0.01% Triton X-100. After washing with PBS with 0.01% Triton X-100, the ABO were stained with DAPI. Images were obtained using a Nikon Eclipse Ti2 fluorescence microscope imaging system. The detailed antibody information is listed in Supplementary Table 1.

### Live staining

Diluted primary antibodies were added in fresh medium to the ABO gently. Incubate the ABO in a 5% CO2 incubator at 37°C for 40 min. Wash the ABO once using fresh medium before adding the diluted secondary antibody in fresh medium and incubate the ABO in a 5% CO2 incubator at 37°C for 40 min. Wash the ABO. Imaging the stained ABO using Nikon Eclipse Ti2 fluorescence microscope imaging system. The detailed antibody information is listed in Supplementary Table 1.

### Microglia phagocytosis assay

The phagocytic activity of iPSC-derived microglia was examined by live imaging using Zeiss Observer Z1 microscope. Microglia (1 × 10^4^ cells/well) were seeded into a Matrigel-coated 96-well plate. Microglia were treated with 10 µg/ml pHrodo red-labeled human synaptosomes at 37°C (5% CO_2_ with 100% humanity) in the Pecon incubation system associated with the Zeiss Observer Z1 microscope. Microglial phagocytosis was evaluated after incubation with the pHrodo-labeled human synaptosome within 6 h.

### Statistics and reproducibility

Statistical analysis was carried out by using GraphPad Prism 10. Analysis of variance (ANOVA) or unpaired *t*-test was used for statistical analysis as indicated in each figure legend. A *p*-value of <0.05 was considered statistically significant. The sample size and replicates of each experiment are indicated in the figure legends. Data are presented as mean with standard deviation (SD) or standard error (SE) as specified in the figure legends.

### Reporting summary

Further information on research design is available in the Nature Portfolio Reporting Summary linked to this article.

## Supplementary information

Supplementary Information

Description of Additional Supplementary Materials

Supplementary Data 1

Reporting Summary

## Data Availability

The primary data supporting the results in this study are available within the paper and its Supplementary Information and Supplementary Data 1. No RNA-seq data or code are included in this study. All data supporting the findings of this study are available from the corresponding authors upon reasonable request.
