# Supplementary material for: Human iPSC-derived microglial cells protect neurons from neurodegeneration in long-term cultured adhesion brain organoids
Source: Commun Biol. 2025 Jan 9;8:30. doi: 10.1038/s42003-024-07401-0 (PMC11718079; doi:10.1038/s42003-024-07401-0)
Supplement: Supplementary file 1 — Supplementary Information [file 42003_2024_7401_MOESM1_ESM.pdf]

Human iPSC-derived microglial cells protect neurons from neurodegeneration in long-term cultured adhesion brain organoids

Xianwei Chen[1,2,3], Guoqiang Sun[1,3], Lizhao Feng[1], E Tian[1], Yanhong Shi[1,]*

[1]Department of Neurodegenerative Diseases, Beckman Research Institute of City of Hope, 1500 E. Duarte Rd, Duarte, CA 91010, USA

[2]State Key Laboratory of Cardiovascular Diseases and Medical Innovation Center, Shanghai East Hospital, School of Life Sciences and Technology, Frontier Science Center for Stem Cell Research, Tongji University, Shanghai 200092, China

[3]Equal contribution.

*Correspondence: yshi@coh.org (Y.S.)

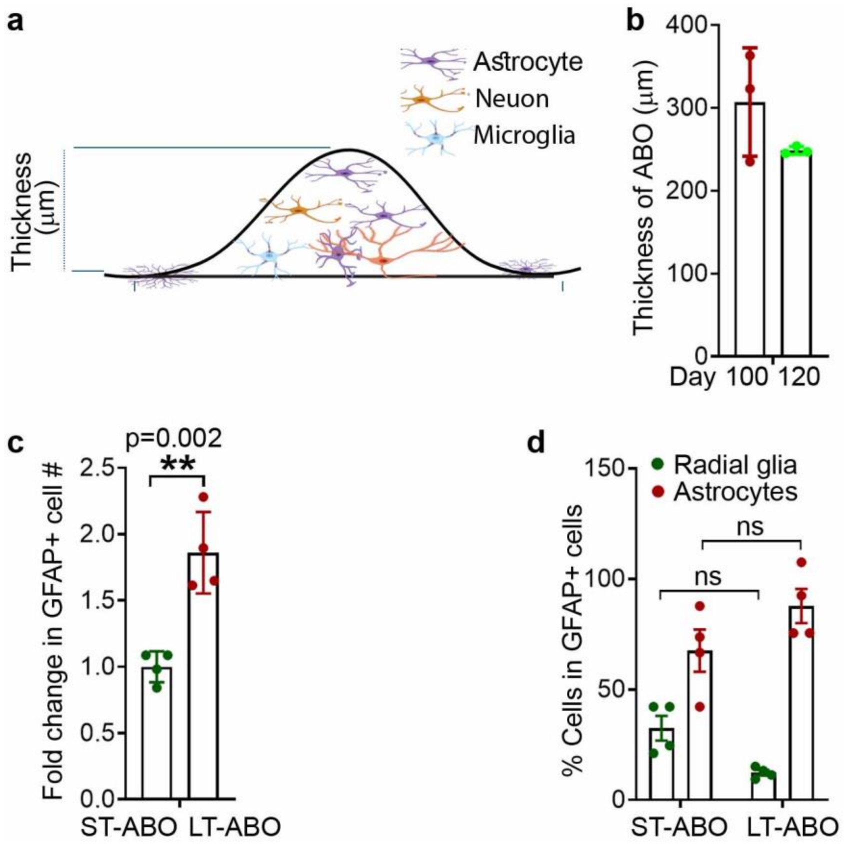

Supplementary Figure 1 Characterization of ABO. a, A schematic showing the shape of the ABO. **b**, The thickness of the core of the ABO. n=3 organoids for each stage. Error bars are SD of the mean. **c**, The fold change in $GFAP^+$ cells in the outer region of the ST-ABO and the LT-ABO. n=4 organoids for the ST-ABO and the LT-ABO, respectively. Error bars are SD of the mean; **p<0.01 by unpaired t-test. **d**, The percentage of the $SOX2^-GFAP^+$ astrocytes and the $SOX2^+GFAP^+$ radial glial cells in $GFAP^+$ cells in the outer region of the ST-ABO and the LT-ABO. n=4 organoids for the ST-ABO and the LT-ABO, respectively. Error bars are SEM of the mean; ns: p>0.05, not statistically significant by two-way ANOVA followed by Tukey's multiple comparison test.

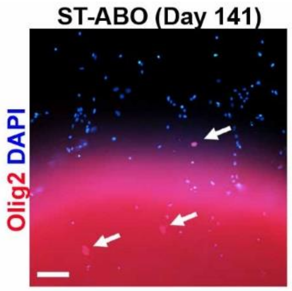

Supplementary Figure 2 Differentiation of OPC in the ST-ABO. Representative image of Olig2 staining in the ST-ABO at day 141 of organoid differentiation. Scale bar, 50 μm.

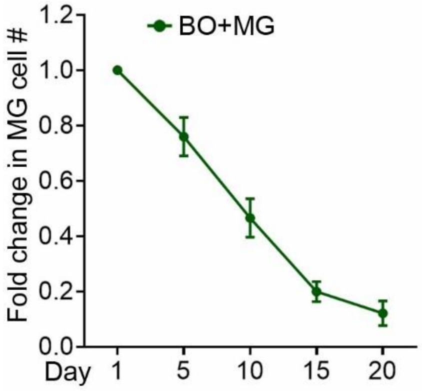

Supplementary Figure 3 Co-culture of microglia with the conventional brain organoids (BO). The fold change of microglia (MG) cell number (#) in the co-culture with the conventional BO for 20 days. n=3 organoids for each time point. Error bars are SD of the mean.

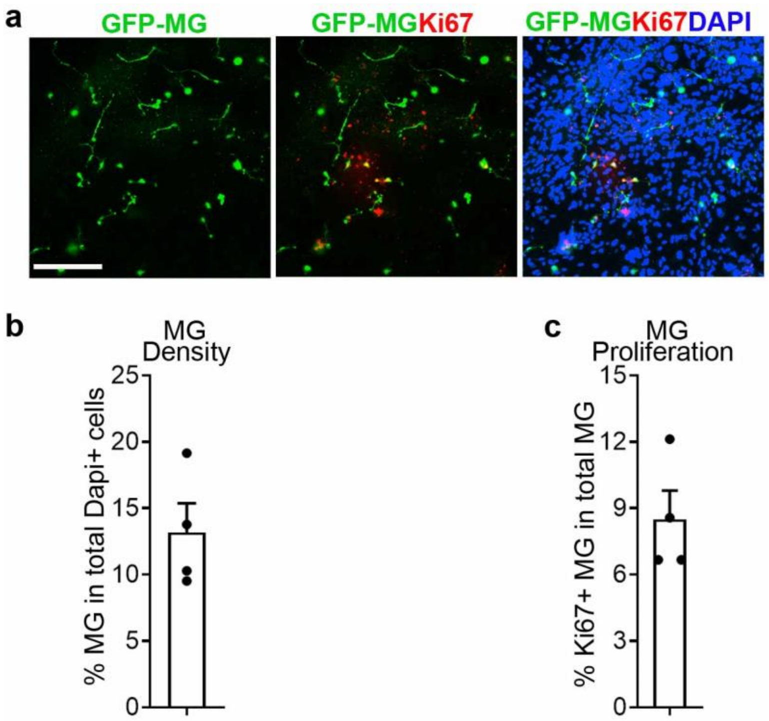

Supplementary Figure 4 Microglial status in the LT-ABO. a, Imaging of the LT-MG-ABO for the GFP-labeled microglia (GFP-MG) and the proliferative marker Ki67 immunostaining. The merged image with DAPI counterstaining is shown on the right. **b, c**, The density (% of GFP-MG cells in total Dapi+ cells, b) and proliferative rate (% of $Ki67^{+}$ GFP-MG cells in total GFP-MG cells, c) of microglia co-cultured with the LT-ABO. n=4 organoids. Error bars are SD of the mean.

Supplementary Table 1 The antibodies used in this study.

Antibodies	Vender	Catalog number
Goat polyclonal anti-SOX2	R&D	AF2018
Mouse monoclonal anti-TUJ1	Covance	PRB-435P
Rat monoclonal anti-CTIP2	abcam	ab18465
Rabbit monoclonal anti-TBR2	abcam	ab216870
Rabbit polyclonal anti-Olig2	Millipore	AB9610
Mouse monoclonal anti-O4 (IgM)	sigma	O7139
Mouse monoclonal anti-MOG	Millipore	MAB5680
Goat polyclonal anti-SOX10	R&D systems	AF2864-SP
Rat monoclonal anti-MBP	Millipore	MAB386
Chicken polyclonal anti-MAP2	abcam	ab5392
Mouse monoclonal anti-GFAP	Sigma	G3893
Goat polyclonal anti-IBA1	abcam	ab5076
Rabbit monoclonal anti-TREM2	abcam	ab209814
Mouse monoclonal anti-p-Tau (AT8)	Invitrogen	MN1020
Rabbit polyclonal anti-SYN1	synaptic system	106 103
Rabbit polyclonal anti-cleaved caspase 3 (c-Cas3)	Cell Signaling	9661S
Rabbit monoclonal anti-Ki67	Lab vision	RM-9106
