# Supplementary material for: Human iPSC-derived microglial cells protect neurons from neurodegeneration in long-term cultured adhesion brain organoids
Source: Commun Biol. 2025 Jan 9;8:30. doi: 10.1038/s42003-024-07401-0 (PMC11718079; doi:10.1038/s42003-024-07401-0)
Supplement: Supplementary file 4 — Reporting Summary [file 42003_2024_7401_MOESM4_ESM.pdf]

Corresponding author(s): Yanhong Shi

Last updated by author(s): Dec 8, 2024

Reporting Summary

Nature Portfolio wishes to improve the reproducibility of the work that we publish. This form provides structure for consistency and transparency in reporting. For further information on Nature Portfolio policies, see our Editorial Policies and the Editorial Policy Checklist.

Statistics

For all statistical analyses, confirm that the following items are present in the figure legend, table legend, main text, or Methods section.

n/a	Confirmed	
☐	☒	The exact sample size (*n*) for each experimental group/condition, given as a discrete number and unit of measurement
☐	☒	A statement on whether measurements were taken from distinct samples or whether the same sample was measured repeatedly
☐	☒	The statistical test(s) used AND whether they are one- or two-sided<br>*Only common tests should be described solely by name; describe more complex techniques in the Methods section.*
☒	☐	A description of all covariates tested
☒	☐	A description of any assumptions or corrections, such as tests of normality and adjustment for multiple comparisons
☐	☒	A full description of the statistical parameters including central tendency (e.g. means) or other basic estimates (e.g. regression coefficient) AND variation (e.g. standard deviation) or associated estimates of uncertainty (e.g. confidence intervals)
☒	☐	For null hypothesis testing, the test statistic (e.g. F, t, r) with confidence intervals, effect sizes, degrees of freedom and P value noted<br>*Give P values as exact values whenever suitable.*
☒	☐	For Bayesian analysis, information on the choice of priors and Markov chain Monte Carlo settings
☒	☐	For hierarchical and complex designs, identification of the appropriate level for tests and full reporting of outcomes
☒	☐	Estimates of effect sizes (e.g. Cohen's *d*, Pearson's *r*), indicating how they were calculated

Our web collection on statistics for biologists contains articles on many of the points above.

Software and code

Policy information about availability of computer code

Data collection	Carl Zeiss LSM700 confocal microscope or Nikon Eclipse Ti2 fluorescence microscope imaging system was used to collect image data. No computer code was used.
Data analysis	Carl Zeiss LSM700 confocal microscope or Nikon Eclipse Ti2 fluorescence microscope imaging system, Graphpad prism and Photoshop software were used for data analysis. No computer code was used.

For manuscripts utilizing custom algorithms or software that are central to the research but not yet described in published literature, software must be made available to editors and reviewers. We strongly encourage code deposition in a community repository (e.g. GitHub). See the Nature Portfolio guidelines for submitting code & software for further information.

Data

Policy information about availability of data

All manuscripts must include a data availability statement. This statement should provide the following information, where applicable:

- Accession codes, unique identifiers, or web links for publicly available datasets
- A description of any restrictions on data availability
- For clinical datasets or third party data, please ensure that the statement adheres to our policy

No Seq data is included in this study.

Human research participants

Policy information about studies involving human research participants and Sex and Gender in Research.

Reporting on sex and gender	n/a
Population characteristics	n/a
Recruitment	n/a
Ethics oversight	n/a

Note that full information on the approval of the study protocol must also be provided in the manuscript.

Field-specific reporting

Please select the one below that is the best fit for your research. If you are not sure, read the appropriate sections before making your selection.

☒ Life sciences ☐ Behavioural & social sciences ☐ Ecological, evolutionary & environmental sciences

For a reference copy of the document with all sections, see nature.com/documents/nr-reporting-summary-flat.pdf

Life sciences study design

All studies must disclose on these points even when the disclosure is negative.

Sample size	Three to four organoids per group were included in this study. Based on Bagley et al. (Nature Methods 2017) and Miura et al, (Nature Biotechnology 2020), including three samples per group allows p value calculation.
Data exclusions	No samples were excluded from analysis.
Replication	All experiments were replicated and the results were consistent.
Randomization	Organoids were randomly selected for experiments.
Blinding	Investigators were not blinded during data acquisition.

Reporting for specific materials, systems and methods

We require information from authors about some types of materials, experimental systems and methods used in many studies. Here, indicate whether each material, system or method listed is relevant to your study. If you are not sure if a list item applies to your research, read the appropriate section before selecting a response.

Materials & experimental systems

n/a	Involved in the study	
☐	☒	Antibodies
☒	☐	Eukaryotic cell lines
☒	☐	Palaeontology and archaeology
☒	☐	Animals and other organisms
☒	☐	Clinical data
☒	☐	Dual use research of concern

Methods

n/a	Involved in the study	
☒	☐	ChIP-seq
☒	☐	Flow cytometry
☒	☐	MRI-based neuroimaging

Antibodies

Antibodies used	Goat polyclonal anti-SOX2, R&D, AF2018; Mouse monoclonal anti-TUJ1, Covance, PRB-435P; Rat monoclonal anti-CTIP2, abcam, ab18465; Rabbit monoclonal anti-TBR2, abcam, ab216870; Rabbit polyclonal anti-Olig2, Millipore, AB9610; Mouse monoclonal anti-O4 (IgM), sigma, O7139; Mouse monoclonal anti-MOG, Millipore, MAB5680; Goat polyclonal anti-SOX10, R&D, AF2864-SP; Rat monoclonal anti-MBP, Millipore, MAB386; Chicken polyclonal anti-MAP2, abcam, ab5392; Mouse monoclonal anti-GFAP, sigma, G3893; Goat polyclonal anti-IBA1, abcam, ab5076; Rabbit monoclonal anti-TREM2, abcam, ab209814; Mouse monoclonal anti-p-Tau (AT8), Invitrogen, MN1020; Rabbit polyclonal anti-SYN1 synaptic system, 106103; Rabbit polyclonal anti-cleaved caspase 3 (c-Cas3), Cell Signaling, 9661S. Rabbit Anti-Human Ki67 (Ki-67) Monoclonal Antibody, Unconjugated, Clone SP6,RM-9106, Lab vision.

Validation	Anti-SOX2 antibody (AF2018) is validated by the manufacturer with 176 citations; anti-TUJ1 antibody (PRB-435P) is validated by the manufacturer with 135 citations; anti-CTIP2 (ab18465) is validated by the manufacturer with 567 citations; anti-TBR2 (ab216870) is validated by manufacturer with 3 citations; anti-Olig2 abtibody (AB9610) is validated by manufacturer with 107 citations; anti-O4 antibody (O7139) is validated by published papers (Li Li et al, Cell Stem Cell, 2018 and 3 more mentioned on the manufacturer's website); anti-MOG antibody (MAB5680) is validated by the manufacturer with 4 citations; anti-SOX10 antibody (AF2864-SP) is validated by the manufacturer with 43 citations; anti-MBP antibody (MAB386) is validated by the manufacturer with 61 citations; anti-MAP2 (ab5392) is validated by the manufacturer with 549 citations; anti-GFAP (G3893) is validated by the manufacturer with 4 citations; anti-IBA1 (ab5076) is validated by the manufacturer with 844 citations; anti-TREM2 (ab209814) is validated by the manufacturer with 5 citations; anti-p-Tau (AT8) antibody is validated by the manufacturer with 737 citations; anti-SYN1 (106 103) is validated by the manufacturer with 37 citations; anti-cleaved caspase 3 (9661S) is validated by the manufacturer with 14003 citations. The citations mentioned on the manufacturer's website are counted above on Jan. 29, 2023; anti-Ki67 (RM-9106) has been validated and cited by 89 citations.
